# Cardiometabolic Treatment Gap in Routine Psychiatric Care: A Cross‐Sectional Study of 1553 Patients

**DOI:** 10.1111/acps.70124

**Published:** 2026-07-07

**Authors:** Vincent Achour, Melanie Faugere, Laurent Boyer, Christophe Lançon, Theo Korchia

**Affiliations:** ^1^ Assistance Publique – Hôpitaux de Marseille Marseille France; ^2^ Aix Marseille Université Marseille France; ^3^ Psynovia: Centre de Recherche en Santé Mentale et Psychiatrie Marseille France

## Abstract

**Background:**

People with severe mental illness (SMI) have elevated cardiometabolic morbidity and mortality, with similar concerns increasingly reported in other psychiatric conditions including anxiety disorders and ADHD. Somatic conditions are often underdiagnosed and pharmacologically undermanaged in this population. We aimed to quantify the alignment between objectively measured cardiometabolic abnormalities and reported pharmacological treatments in a large transdiagnostic psychiatric cohort.

**Methods:**

We conducted a monocentric cross‐sectional observational study in a specialized psychiatric outpatient setting in France between 2018 and 2023. Adults (18–65 years) with schizophrenia, major depressive disorder, bipolar disorder, anxiety disorders, or attention‐deficit and hyperactivity disorder were included. Standardized clinical and laboratory assessments captured blood pressure, fasting plasma glucose, lipid profile, waist circumference, metabolic syndrome, and HbA1c. Somatic medications (antihypertensives, statins, metformin) and self‐reported hypertension/diabetes were recorded. The discordance between measured cardiometabolic abnormalities and reported pharmacological treatments was estimated using a bounding approach within the intersection of available data for each pair of variables.

**Results:**

Among 1553 patients (mean age 38.8 ± 14.2 years; 58.5% women), elevated blood pressure was present in 35.2% whereas 5.7% reported antihypertensive treatment. Hypercholesterolemia was observed in 50.5% while 2.5% reported statin use. High FPG (≥ 5.6 mmol/L) occurred in 12.6%, HbA1c ≥ 48 mmol/mol (≥ 6.5%) in 2.3%, and 1.8% reported metformin treatment. Abdominal obesity was observed in 59.8%, and metabolic syndrome in 20.9%. Applying a bounding approach within complete‐case subsamples, between 29.5% and 35.3% of patients had elevated blood pressure without reporting antihypertensive treatment, between 47.9% and 50.7% had hypercholesterolemia without statins, and between 10.8% and 12.6% had high FPG without metformin.

**Conclusions:**

In this large monocentric psychiatric cohort, cardiometabolic abnormalities were frequent yet poorly reflected in patient‐reported pharmacological treatment. These findings highlight the need to better understand and address the gap between detection and treatment of cardiometabolic conditions in psychiatric care, and to ensure that abnormal findings translate into appropriate clinical follow‐up.

## Highlights

- Significant Outcomes
  ○ In a large transdiagnostic psychiatric cohort, cardiometabolic abnormalities were frequent, with elevated blood pressure in about one‐third and hypercholesterolemia in about half of patients.
  ○ A substantial discordance was observed between measured cardiometabolic abnormalities and reported pharmacological treatment, with only a small minority of affected patients reporting the corresponding antihypertensive, lipid‐lowering, or antidiabetic treatment.
  ○ The burden of measured cardiometabolic abnormalities was highest in schizophrenia and increased with the number of concurrently prescribed antipsychotics, while the treatment–prevalence discordance was observed across the diagnostic spectrum.
- Limitations
  ○ The cross‐sectional, single‐center design limits causal interpretation and the generalizability of the findings.
  ○ Information on nonpsychiatric diagnoses and their treatments relied on patient self‐report, which may be subject to misclassification and does not allow nonprescription to be distinguished from treatment refusal or discontinuation.
  ○ Antidiabetic treatment was assessed through metformin only, and the discordance estimates could not be reliably stratified by individual diagnostic group owing to the limited number of patients reporting each somatic treatment.

**Abbreviations**
AD: anxiety disorder
ADHD: attention deficit/hyperactivity disorder
BD: bipolar disorder
BMI: body mass index
CGI: clinical global impression
DSM‐5: diagnostic and statistical manual of mental disorders, 5th edition
FPG: fasting plasma glucose
GAF: global assessment of functioning
HbA1c: glycated hemoglobin (hemoglobin A1c)
HDL: high‐density lipoprotein (cholesterol)
IDF: International Diabetes Federation
LDL: low‐density lipoprotein (cholesterol)
MARS: Medication Adherence Rating Scale
MDD: major depressive disorder
NSAID: nonsteroidal anti‐inflammatory drug
PRL: prolactin
PTSD: post‐traumatic stress disorder
SD: standard deviation
SF‐36: 36‐item Short Form Health Survey
SMI: Severe mental illness
SQoL‐18: Schizophrenia Quality of Life questionnaire, 18 items
STAI‐YA: State‐Trait Anxiety Inventory, Form Y‐A
SZ: schizophrenia
UKU: Udvalg for Kliniske Undersøgelser Side Effect Rating Scale

## 1 Introduction

Psychiatric disorders such as schizophrenia (SZ), major depressive disorder (MDD), bipolar disorder (BD), and anxiety disorder (AD) affect millions of individuals worldwide and are associated with profound impairments across multiple domains [1]. These conditions significantly reduce quality of life [2] and generate a substantial economic burden for healthcare systems and society at large [3, 4]. Beyond their mental manifestations, psychiatric disorders—and particularly severe mental illnesses (SMI), which include SZ, BD, and MDD—are increasingly recognized as systemic illnesses that profoundly interact with physical health, particularly cardiometabolic functioning [5]. These disorders are associated with a 15–20‐year reduction in life expectancy compared with the general population [6]. Although suicide mortality is higher among patients with SZ or BD [7], approximately 70% of deaths in this group are attributable to somatic diseases [8]. Up to one third of patients with these psychiatric disorders die from cardiovascular causes [9, 10].

Hypertension is more common among patients with psychiatric disorders than in the general population [11, 12, 13], and when present, it carries a higher risk of cardiovascular death in this group than in unaffected individuals [14]. Furthermore, the risk for sudden cardiac death in patients with SMI is fivefold more common than in the general population [5]. Similarly regarding glucose metabolism, type 2 diabetes is two to three times more frequent in patients with psychiatric disorders, who also experience greater clinical consequences of chronic hyperglycemia [15]. A systematic review has reported an association between dysglycaemia and schizophrenia illness severity, particularly with respect to negative symptoms and cognitive impairment [16]. Overweight status and obesity are also highly prevalent in SMI, with up to half of patients with schizophrenia or bipolar disorder meeting criteria for obesity. Meta‐analytic data suggest a markedly higher prevalence of obesity in adults with ADHD compared with adults without [17], and a two‐fold increased risk of any cardiovascular disease in this population [18]. Results are less consistent for lipid metabolism [19]. Anxiety disorders have similarly been associated with elevated cardiovascular morbidity and mortality [20] and with an increased prevalence of metabolic syndrome [21], although their cardiometabolic management has received less attention than that for schizophrenia or bipolar disorder.

The reasons underlying this excess cardiometabolic burden are multifactorial. Biological mechanisms such as chronic low‐grade inflammation [22] and metabolic side effects of medications [23, 24] contribute to increased cardiovascular and metabolic vulnerability. In addition, behavioral risk factors including a sedentary lifestyle [25, 26] and high rates of tobacco use [27] are also more prevalent in psychiatric populations. One key component for this excess mortality is that patients with mental illness are less likely to be diagnosed and treated pharmacologically for their comorbidities than their counterparts [28, 29], even when they meet objective clinical and biological criteria. A recent scoping review highlighted the absence of a streamlined approach to metabolic monitoring in adults with SMI receiving second‐generation antipsychotics [30], and population‐based data from Canada showed that over one‐third of adults treated with atypical antipsychotics did not receive any recommended metabolic screening, with younger adults being the least likely to be monitored [31]. In the French national FACE‐SZ cohort, the prevalence of metabolic syndrome and abdominal obesity in patients with schizophrenia was elevated, with only 10% of those with hypertension, 18% with high fasting glucose, and 8% with dyslipidemia receiving the corresponding treatment—leading the authors to describe these conditions as “considerably under‐diagnosed and under‐treated” [32].

In this context, it appears crucial to quantify the alignment between cardiometabolic risk profiles and ongoing somatic treatments in patients receiving psychiatric care. The objective of the present study is to evaluate this alignment in a large cohort of adult outpatients attending a French university psychiatric center for the management of SZ, MDD, BD, AD, and ADHD. Specifically, we aim to estimate the prevalence of self‐reported and objectively measured cardiometabolic abnormalities, quantify the proportion of patients receiving appropriate pharmacological treatment, and identify critical gaps between measured medical needs and real‐life therapeutic practices. By providing a detailed assessment of these gaps, our findings may help to inform strategies to improve somatic care and ultimately reduce preventable cardiovascular morbidity and mortality among individuals with psychiatric disorders.

### 1.1 Aims of the Study

This study aimed to characterize the cardiometabolic profile of patients across a broad range of psychiatric disorders treated in a routine specialized setting, and to quantify the discordance between objectively measured cardiometabolic abnormalities and the pharmacological treatments that patients reported receiving.

## 2 Materials and Methods

### 2.1 Study Design and Population

This cross‐sectional observational study was carried out at a specialized psychiatric center in Marseille, France. It included adult patients with a primary diagnosis of schizophrenia, major depressive disorder, bipolar disorder, attention deficit and hyperactivity disorder, or anxiety disorder (general anxiety disorder, agoraphobia, panic disorder, social phobia, or post‐traumatic stress disorder (PTSD)) established according to DSM‐5 [33] criteria. Comorbid psychiatric diagnoses were allowed and systematically recorded; therefore, patients could meet criteria for more than one DSM‐5 disorder. Participants were recruited during their first psychiatric outpatient consultation if they met the inclusion criteria and agreed to participate. Data were collected between January 2018 and June 2023 by three authors (V.A., M.F., and T.K.). The data used in this study are part of a monocentric database, constructed with all the parameters systematically evaluated in patients coming for care or expertise. It includes demographic, clinical, and biological information, allowing the investigation of history of hypertension, diabetes, and obesity.

### 2.2 Inclusion and Exclusion Criteria

Inclusion criteria required participants to be between 18 and 65 years old, to have a diagnosis of SZ, MDD, BD, AD, or ADHD, and to be receiving ongoing treatment or follow‐up at the Sainte Marguerite psychiatric center for these conditions. Sainte‐Marguerite Hospital is a teaching hospital of the *Assistance Publique des Hôpitaux de Marseille* (AP‐HM), the public university hospital of Marseille, France. The psychiatry department is housed in a dedicated building including outpatient consultations, day‐hospital services, full‐time inpatient units (68 beds), and clinical assessment facilities. The clinical staff includes psychiatrists, advanced practice nurses (*infirmiers en pratique avancée*), registered nurses, psychologists, clinical pharmacists, and nursing assistants. Prescriptions are issued by psychiatrists, and somatic care for patients with stable comorbidities is coordinated with the patient's general practitioner outside the hospital. Specialized somatic care can be referred internally to other departments of AP‐HM when needed.

Exclusion criteria, evaluated by the physician, included current pregnancy or plans to become pregnant within 6 months, the presence of severe or unstable chronic medical or neurological illnesses that could interfere with study outcomes (such as autoimmune diseases or cancer), and an inability to complete clinical evaluations due to marked cognitive impairment or acute psychiatric symptomatology. Between 2018 and 2023, 1594 patients were screened for eligibility. After application of the exclusion criteria, withdrawal of consent, and missing data retrieval, 1553 patients were included in the analyses (as mentioned in the flow chart, Figure 1).

**FIGURE 1:**
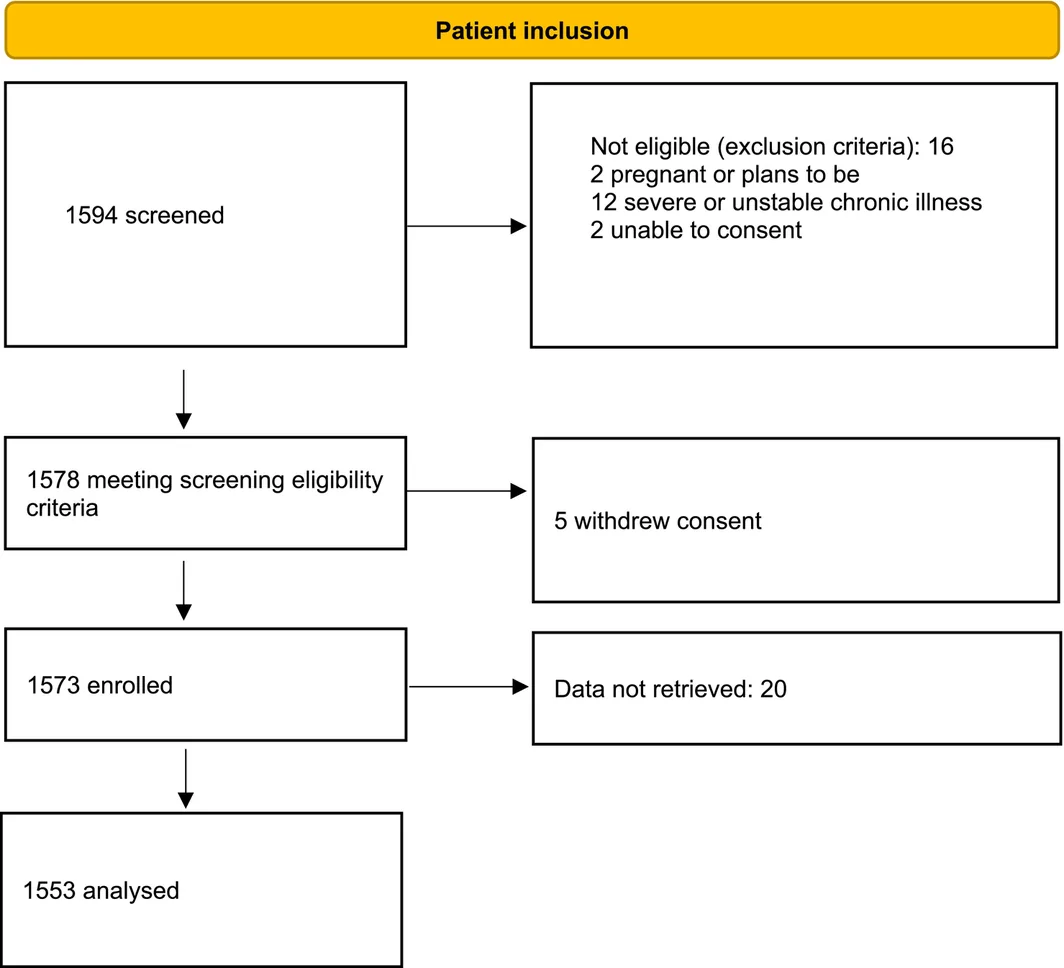
Flow chart.

All the patients only contributed once, during their first evaluation—the index visit—to the database used in the present study. Although they were offered to be evaluated one time every year to establish a monitoring of their physical and mental health.

### 2.3 Data Gathering

#### 2.3.1 Sociodemographic and Clinical Data

Clinical data were collected during medical consultations by trained physicians from medical records and standardized interviews, combining a set of validated, structured psychometric instruments administered according to their standardized procedures and a clinical psychiatric interview conducted by the psychiatrist. Sociodemographic data such as age, sex, employment status (currently working or not working), housing status, and education level were recorded as well as psychiatric and somatic history at the index visit. A history of hypertension and diabetes was systematically investigated. Information on current psychiatric and somatic medications, focusing on the pharmacological classes corresponding to the cardiometabolic parameters assessed in this study, and treatment implicated in the management of adverse effects of psychotropic medication, was obtained through patient self‐report during the clinical interviews (metformin, antihypertensive agents, statins, synthetic thyroid hormones). We did not have access to dispensing or prescription registries. During the consultation, a registered nurse measured patients' weight and height, blood pressure, and abdominal circumference. Measurements were taken once, at the index visit. The patients were also assessed for substance use, including tobacco, alcohol, and cannabis.

#### 2.3.2 Laboratory Investigations

A venous blood sample was collected on a fasting basis using a standardized procedure to measure lipid profile (total cholesterol, LDL‐cholesterol, HDL‐cholesterol, and triglycerides), prolactin levels, and two complementary indicators of glucose metabolism abnormalities, fasting plasma glucose (FPG) and HbA1c. High‐sensitivity C‐reactive protein was measured as a marker of low‐grade inflammation, and Thyroid‐Stimulating Hormone (TSH) was measured as part of the routine biological assessment of patients receiving psychotropic medication, given the potential influence of lithium and several antipsychotics on thyroid function.

#### 2.3.3 Clinical Scales

Several standardized scales were used to better characterize our sample in terms of symptoms, adverse effects, and quality of life. The Global Assessment of Functioning (GAF) [34] was used to evaluate overall functional capacity and remission. Anxiety levels were measured using the State‐Trait Anxiety Inventory (STAI‐YA) [35], while treatment adherence was assessed with the Medication Adherence Rating Scale (MARS) [36]. Quality of life was evaluated using the Short Form Health Survey (SF‐36) [37] and the SQoL‐18 scale [38]. Symptom severity and treatment response were assessed with the Clinical Global Impression Scale (CGI) [39], and side effects were evaluated across 4 domains, psychic, neurological, autonomic and other side effects, using the Udvalg for Kliniske Undersøgelser Side Effect Rating Scale (UKU) [40].

### 2.4 Operational Definitions of Diseases and Abnormalities

The thresholds used to diagnose the abnormalities that we studied are defined according to the 2006 International Diabetes Federation (IDF) criteria [41]. Cutoffs for elevated blood pressure (BP) were systolic BP ≥ 130 mmHg or diastolic BP ≥ 85 mmHg. Abdominal obesity was defined as a waist circumference ≥ 94 cm in men and ≥ 80 cm in women. All measurements were conducted by registered nurses, following standardized protocols [42].

The cutoffs for biological criteria were as follows: high fasting plasma glucose defined as FPG ≥ 5.6 mmol/L, hypertriglyceridemia ≥ 1.7 mmol/L, reduced HDL‐cholesterol < 1.03 mmol/L in men and < 1.29 mmol/L in women (these three parameters as defined by the IDF), elevated total cholesterol ≥ 5.17 mmol/L, high LDL‐cholesterol ≥ 4.14 mmol/L, hyperprolactinemia defined as prolactin levels ≥ 619.9 mIU/L in women and ≥ 375.8 mIU/L in men. HbA1c was analyzed as a categorical variable, using the threshold ≥ 48 mmol/mol (≥ 6.5%) to indicate diabetes‐range glycaemia.

Metabolic syndrome was defined according to the IDF 2006 criteria [41], which require the presence of central obesity as described above combined with at least two additional metabolic abnormalities. These include elevated triglycerides (or ongoing treatment for hypertriglyceridemia), reduced HDL‐cholesterol (or specific treatment for this abnormality), elevated blood pressure (or treatment for previously diagnosed arterial hypertension), and elevated FPG (or a prior diagnosis of type 2 diabetes). For biological parameters not covered by the IDF definition (such as LDL‐cholesterol, total cholesterol, and prolactin), cutoff values were based on the reference ranges provided by our institution's central laboratory, indicated in Tables 1 and 2.

**TABLE 1:** Population characteristics at the index visit.

	*N* = 1553^a^	95% confidence interval
	Mean (SD) or N (%)	
Sociodemographic		
Sex		
Men	644 (41.5%)	39.0–43.9
Women	909 (58.5%)	56.1–61.0
Age (years)	38.83 (14.15)	38.1–39.5
Education level (years) (*n* = 1048)	12.60 (3.05)	12.4–12.8
Education level (> 12 years) (*n* = 1048)	697 (66.5%)	63.6–69.3
Single (*n* = 1515)	934 (61.7%)	59.2–64.1
Professionally active (*n* = 1519)	364 (24.0%)	21.9–26.2
Diagnosis		
Schizophrenia	554 (35.7%)	33.3–38.1
Major depressive disorder	533 (34.3%)	32.0–36.7
Bipolar disorder	161 (10.4%)	8.9–12.0
General anxiety disorder (*n* = 1524)	496 (32.5%)	30.2–34.9
Agoraphobia (*n* = 1524)	223 (14.4%)	12.9–16.5
Panic disorder (*n* = 1524)	290 (19.0%)	17.1–21.1
Social phobia (*n* = 1525)	251 (16.2%)	14.7–18.4
Post traumatic stress disorder (*n* = 1524)	89 (5.7%)	4.8–7.1
Attention deficit hyperactivity disorder (*n* = 1551)	279 (18.0%)	16.2–20.0
Substances use		
Current tobacco smoking (*n* = 1482)	777 (52.4%)	49.9–55.0
Current cannabis consumption (*n* = 1485)	282 (18.9%)	17.1–21.1
Current alcohol abuse (*n* = 1474)	259 (17.6%)	15.7–19.6
Clinical scales		
SQoL‐18^b^ index (*n* = 1013)	50.07 (19.42)	48.9–51.3
SF‐36^c^ mental health score (*n* = 649)	30.54 (13.68)	29.5–31.6
SF‐36 physical health score (*n* = 648)	48.00 (12.31)	47.0–48.9
CGI^d^ (*n* = 924)	4.13 (1.23)	4.1–4.2
GAF^e^ score (*n* = 903)	53.96 (15.87)	52.9–55.0
STAI‐YA^f^ score (*n* = 751)	47.39 (14.02)	46.4–48.4
MARS^g^ score (*n* = 1124)	6.04 (2.35)	5.9–6.2
UKU^h^ I (psychic side effects) (*n* = 626)	5.55 (4.89)	5.2–5.9
UKU II (neurological side effects) (*n* = 625)	1.16 (1.54)	1.0–1.3
UKU III (autonomic side effects) (*n* = 625)	2.81 (2.78)	2.6–3.0
UKU IV (other side effects) (*n* = 625)	3.72 (4.13)	3.4–4.1
Psychotropic treatments		
Chlorpromazine equivalent dose (mg/day)	334.04 (595.73)	304.0–364.1
Antipsychotics (all)	677 (43.6%)	41.1–46.1
Atypical	640 (41.2%)	38.8–43.7
Typical	113 (7.3%)	6.1–8.7
Polypharmacy (at least 2 antipsychotics) (*n* = 1553)	161 (10.4%)	9.0–12.1
Antidepressants	668 (43.0%)	40.6–45.5
Benzodiazepines	411 (26.5%)	24.3–28.7
Mood stabilizers	126 (8.1%)	6.9–9.6
Methyphenidate	45 (2.9%)	2.2–3.9
Somatics		
Body mass index (*n* = 1544)	25.58 (5.78)	25.3–25.9
Obesity (*n* = 1544)	297 (19.1%)	17.3–21.3
Total cholesterol (mmol/L) (*n* = 1439)	5.20 (5.14)	4.9–5.5
hsCRP^i^ (mg/L) (*n* = 1379)	2.07 (2.15)	2.0–2.2
TSH^j^ (mIU/L) (*n* = 1435)	2.21 (1.41)	2.1–2.3
Prolactin (mIU) (*n* = 1164)		
Men	373.20 (428.93)	341.1–405.5
Women	547.19 (838.42)	472.7–623.1

^a^ Patients were recruited exclusively from outpatient psychiatric services.

^b^ Schizophrenia Quality of Life—18 items.

^c^ 36‐Item Short Form Health Survey Questionnaire.

^d^ Clinical Global Impression.

^e^ Global Assessment of Functioning.

^f^ State‐Trait Anxiety Inventory—YA form.

^g^ Medication Adherence Rating Scale.

^h^ Udvalg for Kliniske Undersøgelser.

^i^ High‐sensitivity C‐reactive protein.

^j^ Thyroid‐stimulating hormone.

**TABLE 2:** Reported somatic medications and conditions, and measured cardiometabolic parameters.

	*N* (%)	95% CI
Reported somatic medications		
Metformin (*n* = 1540)	28 (1.8%)	1.3–2.6
Antihypertensive (*n* = 1541)	88 (5.7%)	4.7–7.0
Statins (*n* = 1542)	39 (2.5%)	1.9–3.4
Acetylsalicylic acid (*n* = 1541)	27 (1.7%)	1.2–2.5
Levothyroxine (*n* = 1541)	62 (4.0%)	3.2–5.1
Reported somatic conditions		
High blood pressure (*n* = 1534)	114 (7.3%)	6.2–8.9
Diabetes (*n* = 1534)	66 (4.2%)	3.4–5.4
Measured cardiometabolic parameters		
High blood pressure (systolic BP^a^ ≥ 130 or diastolic BP^a^ ≥ 85 mmHg) (*n* = 1541)	546 (35.2%)	33.1–37.9
Hyperglycemia (fasting plasma glucose ≥ 5.6 mmol/L) (*n* = 1500)	189 (12.6%)	11.0–14.4
Hypertriglyceridemia (≥ 1.7 mmol/L) (*n* = 1488)	314 (21.1%)	19.1–23.2
Low HDL^b^ (< 1.03 mmol/L in men; < 1.29 mmol/L in women) (*n* = 1471)	331 (22.5%)	20.4–24.7
Hypercholesterolemia (≥ 5.17 mmol/L) (*n* = 1439)	727 (50.5%)	47.9–53.1
Abdominal obesity (waist circumference ≥ 94 cm in men; ≥ 80 cm in women) (*n* = 1468)	878 (59.8%)	57.3–62.3
Metabolic Syndrome (*n* = 1481)	309 (20.9%)	18.9–23.0
HbA1C^c^ ≥ 48 mmol/mol (*n* = 851)	20 (2.3%)	1.5–3.6
Hyperprolactinemia men (≥ 375.8 mIU/L) (*n* = 689)	161 (23.4%)	20.4–26.7
Hyperprolactinemia women (≥ 619.9 mIU/L) (*n* = 475)	111 (23.4%)	19.8–27.4

^a^ Blood pressure.

^b^ High density lipoprotein cholesterol.

^c^ Glycated hemoglobin.

### 2.5 Ethics and Consent

All stages of database operation, including data collection and analysis, were approved by the committee responsible for our institution's health data access portal (reference PADS‐N2THY6). The study was conducted in accordance with the Declaration of Helsinki and with French regulations on the use of clinical data for research purposes, including the General Data Protection Regulation (GDPR). All patients provided written informed consent for participation and for the use of their pseudonymized clinical data for research purposes.

### 2.6 Estimation of the Discordance Between Measured Cardiometabolic Abnormalities and Reported Pharmacological Treatment

Information on cardiometabolic parameters and reported somatic medications was available for varying proportions of the cohort, although most variables were available for over 92% of patients. To handle the missing data pattern without imputation, the analysis of discordance between somatic abnormalities and declared treatment was performed on a complete‐case basis within each pair of variables. For each pair (measured abnormality *A*, corresponding pharmacological treatment *T*), the analysis was restricted to the subsample of patients with non‐missing information on both *A* and *T* (denoted *N*_A,T_). Within *N*_A,T_, the lower bound of the proportion of patients with the abnormality and not reporting the corresponding treatment was computed as (*A* − *T*)/*N*_A,T_, corresponding to the assumption that every treated patient also met criteria for the abnormality at the index visit. The upper bound was computed as *A*/*N*_A,T_, corresponding to the assumption that none of the treated patients met criteria for the abnormality at the index visit. This approach was applied to three pairs: elevated blood pressure/antihypertensive medication, hypercholesterolemia/statins, and high fasting plasma glucose/metformin.

### 2.7 Statistical Analysis

Statistical analyses were performed using Jamovi software. All variables were presented using means and dispersion (standard deviation) for continuous variables and frequency distribution for categorical variables. Comparisons between individuals with and without the characteristic described were made using the chi‐squared test for categorical variables and Student's *t*‐test for continuous variables. 95% confidence intervals of the prevalences were calculated using the Wilson method, and Cramér's *V* was used to determine the magnitude of the association. As each patient contributed a single set of measurements obtained at the index visit, observations were independent and no adjustment for repeated measures was required.

## 3 Results

Of the 1553 patients initially included, sample characteristics, treatment data and gathered somatic measurements were available for varying proportions of the cohort depending on the variable; effective denominators for each variable are reported in Tables 1 and 2, and all percentages presented below are calculated within the corresponding available subsamples. When not specified, the denominator is the total sample (*N* = 1553).

### 3.1 Sample Characteristics

A total of 1553 patients were included in our study, with 909 (58.5%) women and 644 (41.5%) men and a mean age of 38.83 years (SD = 14.15 years). Among them, 364 (24.0%) were professionally active, and 934 (61.7%) were single. 554 (35.7%) were diagnosed with schizophrenia, 533 (34.3%) with major depressive disorder, and 161 (10.4%) with bipolar disorder. Anxiety disorders were highly prevalent, with 496 patients (32.5%) meeting criteria for generalized anxiety disorder, 223 (14.4%) for agoraphobia, 290 (19.0%) for panic disorder, 251 (16.2%) for social phobia, and 89 (5.7%) for PTSD. 279 (18.0%) patients were diagnosed with ADHD.

In terms of symptom severity, functioning, quality of life and adverse effects, the cohort exhibited a moderate overall clinical burden. The mean CGI score was 4.13 (SD = 1.23) and the mean GAF score was 53.96 (SD = 15.87), indicating moderate symptom severity and functional impairment. Quality of life was reduced, with a mean SQoL‐18 index of 50.07 (SD = 19.42) on a 0–100 scale (higher scores indicating better quality of life). Health‐related quality of life assessed with the SF‐36 (mental and physical component summaries, normed to a general population mean of 50 with SD = 10) revealed substantially impaired mental health (30.54, SD = 13.68) and near‐normal physical health (48.00, SD = 12.31). Anxiety symptoms were substantial, as reflected by a mean STAI‐YA score of 47.39 (SD = 14.02). Treatment adherence, assessed with the MARS scale (range 0–10, higher scores indicating better adherence), averaged 6.04 (SD = 2.35), corresponding to moderate adherence in the cohort. Finally, self‐reported side effects measured by the UKU showed a moderate adverse‐effect burden across all domains, with a mean score of 5.55 (SD = 4.89) on the psychic (UKU‐I) side effects subscale, 1.16 (SD = 1.54) on the neurological (UKU‐II) subscale, 2.81 (SD = 2.78) on the autonomic (UKU‐III) subscale, and 3.72 (SD = 4.13) on the other side effects subscale (UKU‐IV). Sample characteristics are described in Table 1.

### 3.2 Cardiometabolic Risk Factors

More than half of the sample were smoking tobacco (52.4%), and 282 (18.9%) reported using cannabis regularly. 259 patients (17.6%) met criteria for alcohol abuse. The mean body mass index (BMI) was 25.58 (SD = 5.78) and obesity was observed in 297 patients (19.1%).

### 3.3 Psychiatric Treatment

Regarding psychotropic medication, 677 patients (43.6%) were treated with antipsychotics, with a mean chlorpromazine‐equivalent dose of 334.04 mg/day (SD = 595.73). Most of these prescriptions involved atypical antipsychotics (640 patients, 41.2%), while 113 patients (7.3%) received typical agents. Antipsychotic polypharmacy (i.e., taking 2 or more antipsychotics) was found in 161 patients (10.4%). In addition, 668 patients (43.0%) were taking antidepressants, 411 (26.5%) benzodiazepines, 126 (8.1%) mood stabilizers, and 45 (2.9%) were taking methylphenidate.

### 3.4 History of Arterial Hypertension and Diabetes

114 patients (7.3%) had a diagnosis of high blood pressure, and 66 (4.2%) had a diagnosis of diabetes.

### 3.5 Reported Somatic Treatment

Regarding treatments prescribed in our sample, 88 patients (5.7%) reported receiving antihypertensive medication, 28 (1.8%) were treated with metformin, and 39 (2.5%) with statins. In addition, 62 patients (4.0%) were treated with levothyroxine. Prevalences of cardiovascular abnormalities and reported treatments are shown in Figure 2.

**FIGURE 2:**
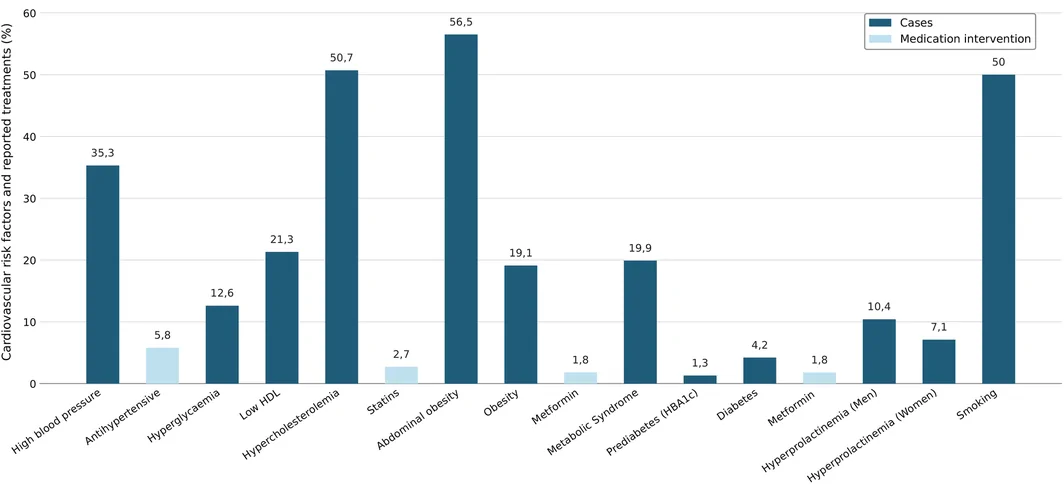
Cardiovascular risk factors and reported treatments.

### 3.6 Measured Somatic Abnormalities

Regarding somatic parameters, 546 patients (35.2%) presented with high blood pressure and 189 (12.6%) with high FPG at the index visit. HbA1c was measured in 851 patients (54.8% of the cohort) and was ≥ 48 mmol/mol in 20 of them (2.3% of those tested). Lipid abnormalities were frequent, with hypertriglyceridemia observed in 314 patients (21.1%), low HDL‐cholesterol in 331 (22.5%), and hypercholesterolemia in 727 (50.5%). Abdominal obesity was present in 878 patients (59.8%), and 309 (20.9%) met criteria for metabolic syndrome. Prolactin was documented in 1164 patients (75.0% of the cohort) and hyperprolactinemia was found in 161 men (23.4% of those tested) and 111 women (23.4%). Reported somatic medications and conditions, and measured cardiometabolic parameters are fully described in Table 2.

### 3.7 Discordance Between Measured Cardiometabolic Abnormalities and Reported Pharmacological Treatment

Three patterns of discordance between measured cardiometabolic abnormalities and reported pharmacological treatment were observed. For each pair, the analysis was restricted to patients with complete information on both the measured parameter and the corresponding treatment (*N*_A,T_). As treatment proportions in this section are computed within *N*_A,T_, they may differ slightly from those reported in Section 3.5, which are based on the full sample with available treatment data. Results are available in Table 3.

**TABLE 3:** Complete‐case subsample (both variables available), *N* (% of total cohort).

Abnormality/treatment pair	*N*_A,T_ (% of the cohort)	Abnormality^a^, *n* (% of *N*_A,T_)	Treatment^a^, *n* (% of *N*_A,T_)	Discrepancy range^b^
Elevated blood pressure/antihypertensive	1529 (98.5%) 97.7–99.0	539 (35.3%) 32.9–37.7	88 (5.8%) 4.7–7.0	**29.5%–35.3%**
Hypercholesterolemia/statins	1429 (92.0%) 90.6–93.3	724 (50.7%) 48.1–53.3	39 (2.7%) 2.0–3.7	**47.9%–50.7%**
High FPG/metformin	1488 (95.8%) 94.7–96.7	188 (12.6%) 11.0–14.4	27 (1.8%) 1.3–2.6	**10.8%–12.6%**

^a^ Prevalences in this table are computed within the complete‐case subsample (*N*_A,T_) and may differ slightly from those in Table 1, which are based on all patients with available data for each variable.

^b^ Bold values indicate the estimated discrepancy range between measured cardiometabolic abnormalities and reported pharmacological treatment.

Elevated blood pressure and antihypertensive treatment (*N*_A,T_ = 1529): within this subsample, elevated blood pressure was measured in 539 patients (35.3%), whereas 88 patients (5.8%) reported antihypertensive treatment. Applying the bounding approach described in the Methods section, between 29.5% and 35.3% of patients in this subsample had elevated blood pressure without reporting antihypertensive treatment.

Hypercholesterolemia and statins (*N*_A,T_ = 1429): within this subsample, hypercholesterolemia was identified in 724 patients (50.7%), whereas 39 patients (2.7%) reported statin use. Between 47.9% and 50.7% of patients had hypercholesterolemia without reporting statin treatment.

High fasting plasma glucose and metformin (*N*_A,T_ = 1488): within this subsample, fasting plasma glucose was high in 188 patients (12.6%), whereas 27 patients (1.8%) reported metformin use. Between 10.8% and 12.6% had high fasting plasma glucose without reporting metformin treatment.

### 3.8 Variations of Measured Cardiometabolic Abnormalities Across Subgroups

Post hoc exploratory analyses revealed substantial heterogeneity in the prevalence of measured cardiometabolic abnormalities across clinical and demographic strata (Table 4). The prevalence of elevated blood pressure, hyperglycemia and metabolic syndrome increased markedly across age tertiles (e.g., elevated blood pressure: 25.4%, 33.6% and 51.3% in the youngest, middle and oldest tertiles, respectively; all *p* < 0.001), in line with established epidemiology in the general population.

**TABLE 4:** Group analysis.

	High blood pressure	(p) magnitude^c^	Hyper glycaemia	(p) magnitude^c^	Hyper triglyceridemia	(p) magnitude^c^	Hypo HDL	(p) magnitude^c^	Abdominal obesity	(p) magnitude^c^	Metabolic syndrome	(p) magnitude^c^
Sex												
Men (vs. women)	353 (39.22%)	—	121 (13.78%)	—	237 (27.24%)	—	188 (21.89%)	—	463 (53.28%)	—	203 (23.39%)	—
Women (vs. men)	193 (30.11%)	< 0.001 0.094	68 (10.93%)	0.101 0.042	77 (12.46%)	< 0.001 0.179	143 (23.37%)	0.503 0.018	415 (69.28%)	< 0.001 0.160	106 (17.29%)	0.004 0.074
Age groups^a^												
Age group 1 (≤ 27.5yo^b^)	105 (25.42%)	< 0.001 0.127	18 (4.49%)	< 0.001 0.148	53 (13.32%)	< 0.001 0.115	92 (23.29%)	0.660 0.012	140 (35.53%)	< 0.001 0.300	40 (10.00%)	< 0.001 0.163
Age group 2 (27.5–50.2 yo^b^)	260 (33.55%)	0.120 0.040	89 (11.74%)	0.311 0.026	182 (24.20%)	0.003 0.077	191 (25.67%)	0.003 0.077	477 (64.55%)	< 0.001 0.097	170 (22.82%)	0.063 0.048
Age group 3 (≥ 50.2 yo^b^)	181 (51.27%)	< 0.001 0.181	82 (24.05%)	< 0.001 0.187	79 (23.37%)	0.245 0.030	48 (14.46%)	< 0.001 0.104	261 (77.91%)	< 0.001 0.201	99 (29.46%)	< 0.001 0.115
Socioeconomics												
Professionally active	116 (31.96%)	0.102 0.042	41 (11.82%)	0.659 0.012	56 (16.14%)	0.009 0.069	68 (19.65%)	0.136 0.039	193 (57.10%)	0.229 0.032	55 (16.03%)	0.012 0.066
Independent housing	393 (39.07%)	< 0.001 0.101	152 (15.48%)	< 0.001 0.121	214 (21.93%)	0.341 0.025	210 (21.72%)	0.262 0.030	628 (65.55%)	< 0.001 0.161	229 (25.58%)	< 0.001 0.092
Single	303 (32.65%)	0.003 0.075	88 (9.64%)	< 0.001 0.111	193 (21.35%)	0.839 0.005	225 (25.22%)	0.002 0.082	487 (54.97%)	< 0.001 0.128	170 (18.85%)	0.019 0.062
Education level (≥ 12 years)	233 (33.53%)	0.538 0.019	77 (11.24%)	0.815 0.007	122 (17.91%)	0.046 0.062	121 (17.98%)	0.020 0.073	369 (55.41%)	0.090 0.054	117 (17.23%)	0.080 0.055
Diagnostics												
SZ (0 vs. 1)	214 (38.91%)	0.035 0.054	67 (12.20%)	0.720 0.009	138 (25.23%)	0.003 0.077	157 (29.24%)	< 0.001 0.122	336 (61.76%)	0.251 0.030	140 (25.69%)	< 0.001 0.090
MDD (0 vs. 1)	195 (36.79%)	0.004 0.092	71 (13.79%)	0.658 0.033	98 (19.33%)	0.004 0.094	91 (18.06%)	< 0.001 0.125	314 (62.18%)	0.003 0.097	102 (20.24%)	0.001 0.105
BD (0 vs. 1)	56 (35.22%)	0.953 0.002	15 (10.07%)	0.326 0.025	35 (23.49%)	0.451 0.020	32 (21.92%)	0.859 0.005	93 (62.84%)	0.428 0.021	27 (18.12%)	0.385 0.023
ADHD (0 vs. 1)	69 (25.18%)	< 0.001 0.100	19 (7.42%)	0.006 0.071	28 (11.02%)	< 0.001 0.112	40 (15.75%)	0.004 0.074	109 (42.41%)	< 0.001 0.164	22 (8.76%)	< 0.001 0.135
General anxiety disorder (0 vs. 1)	166 (33.74%)	0.283 0.028	61 (12.53%)	0.940 0.002	90 (18.60%)	0.105 0.042	100 (20.79%)	0.320 0.026	278 (59.15%)	0.652 0.012	88 (18.22%)	0.092 0.044
Agoraphobia (0 vs. 1)	83 (37.56%)	0.521 0.017	25 (11.57%)	0.639 0.012	53 (24.42%)	0.198 0.034	53 (24.42%)	0.439 0.020	129 (61.14%)	0.728 0.009	48 (22.22%)	0.586 0.014
Panic disorder (0 vs. 1)	97 (33.56%)	0.412 0.021	26 (9.32%)	0.071 0.047	62 (22.22%)	0.619 0.013	70 (25.18%)	0.217 0.032	170 (61.59%)	0.562 0.015	53 (19.06%)	0.420 0.021
Social anxiety disorder (0 vs. 1)	89 (35.46%)	0.953 0.002	32 (13.06%)	0.816 0.006	46 (18.70%)	0.309 0.027	55 (22.63%)	0.920 0.003	139 (58.90%)	0.702 0.010	50 (20.41%)	0.862 0.005
PTSD (0 vs. 1)	28 (31.82%)	0.440 0.020	12 (13.79%)	0.718 0.009	14 (16.28%)	0.256 0.030	24 (28.24%)	0.184 0.035	48 (57.83%)	0.670 0.011	16 (19.05%)	0.678 0.011
Number of antipsychotics taken (typical or atypical)												
None	296 (34.06%)	0.201 0.033	103 (12.34%)	0.729 0.009	137 (16.61%)	< 0.001 0.123	140 (17.16%)	< 0.001 0.145	452 (56.01%)	0.001 0.086	137 (16.67%)	< 0.001 0.115
1 antipsychotic	184 (35.87%)	—	63 (12.45%)	—	134 (26.48%)	—	148 (29.66%)	—	313 (62.10%)	—	129 (25.70%)	—
2 antipsychotics	56 (39.72%)	—	22 (15.60%)	—	39 (28.06%)	—	38 (27.94%)	—	99 (58.24%)	—	39 (28.06%)	—
3 antipsychotics or more	10 (55.56%)	0.200 0.062	1 (5.56%)	0.725 0.037	4 (22.22%)	< 0.001 0.125	5 (29.41%)	< 0.001 0.147	14 (82.35%)	< 0.001 0.115	4 (22.22%)	< 0.001 0.118
Typical	12 (34.29%)	—	5 (14.29%)	—	10 (27.78%)	—	8 (22.22%)	—	27 (75.00%)	—	7 (20.00%)	—
Atypical	204 (36.23%)	—	71 (12.77%)	—	147 (26.49%)	—	161 (29.54%)	—	342 (62.20%)	—	145 (26.27%)	—
Typical and atypical	34 (45.95%)	0.250 0.064	10 (13.51%)	0.955 0.012	20 (27.78%)	0.962 0.011	22 (30.99%)	0.612 0.039	57 (78.08%)	0.010 0.118	20 (27.78%)	0.674 0.035
Baseline BMI												
Obesity (BMI > 30) (vs. BMI < 30)	158 (53.74%)	—	76 (26.12%)	—	111 (38.81%)	—	122 (43.26%)	—	280 (100%)	—	138 (48.76%)	—
BMI < 30 (vs. obesity)	385 (31.00%)	< 0.001 0.187	113 (9.39%)	< 0.001 0.199	202 (16.89%)	< 0.001 0.212	208 (17.57%)	< 0.001 0.242	594 (50.17%)	< 0.001 0.400	171 (14.33%)	< 0.001 0.333

^a^ Groups were defined according to tertiles of the age distribution.

^b^ Years old.

^c^ Magnitude measured by Cramér's V.

Across diagnostic categories, patients with schizophrenia displayed the highest cardiometabolic burden, with significantly elevated prevalences of low HDL‐cholesterol (29.2%, *p* < 0.001), hypertriglyceridemia (25.2%, *p* = 0.003) and metabolic syndrome (25.7%, *p* < 0.001) compared with the rest of the cohort. Patients with ADHD displayed significantly lower prevalences across all components, including elevated blood pressure (25.2%, *p* < 0.001), hyperglycemia (7.4%, *p* = 0.006), hypertriglyceridemia (11.0%, *p* < 0.001), abdominal obesity (42.4%, *p* < 0.001) and metabolic syndrome (8.8%, *p* < 0.001). No significant differences from the rest of the cohort were observed for patients with bipolar disorder or across the anxiety disorder subtypes.

Cardiometabolic burden was also lower in patients not receiving antipsychotic medication: those without any antipsychotic showed significantly lower prevalences of low HDL‐cholesterol (17.2%, *p* < 0.001), hypertriglyceridemia (16.6%, *p* < 0.001), abdominal obesity (56.0%, *p* = 0.001) and metabolic syndrome (16.7%, *p* < 0.001) than the rest of the cohort. Typical and atypical co‐treatment was significantly associated with higher prevalence of abdominal obesity. Effect sizes for these associations, measured by Cramér's V, were generally small, the largest being observed for baseline obesity status (e.g., Cramér's *V* = 0.40 for abdominal obesity and 0.33 for metabolic syndrome) and for the oldest age tertile.

## 4 Discussion

Our study indicates a high prevalence of cardiometabolic disorders among a large transdiagnostic cohort, as well as a substantial gap between the conditions reported by patients, objectively measured parameters, and reported pharmacological treatment. Approximately one‐third of the study population had elevated blood pressure but was not receiving antihypertensive treatment. Hypercholesterolemia was identified in half of our sample, yet only 2.5% reported statin use. These findings are consistent with a large body of literature showing that cardiometabolic risk is markedly elevated in people with psychiatric disorders [43], particularly those with SMI [44, 45]. Several meta‐analyses have shown a high prevalence of metabolic syndrome in major psychiatric disorders, including MDD, SZ, and BD [46, 47, 48]. However, our study does not only confirm an increased cardiometabolic risk: It also quantifies a substantial mismatch between measured somatic needs and real‐life pharmacological management, highlighting a three‐level discrepancy between what patients report, what we measure, and what they take as treatment.

In the general population, international data suggest that a substantial proportion of individuals with cardiometabolic disease do receive pharmacological treatment, even though care remains far from optimal. For example, pooled analyses from 1201 studies indicate that globally around 47% of women and 38% of men with hypertension are treated, with treatment rates in many high‐income countries reaching 60%–80% of all hypertensive adults [49]. Similarly, in a nationally representative US sample, approximately one‐third of adults meet guideline‐based indications for statin therapy and about 47% of those for whom statins are recommended receive them [50]. On a global scale, recent estimates show that among people with diabetes, more than half are diagnosed and over 90% of those with a diagnosis are treated with glucose‐lowering medication [51].

By contrast, only a small minority of patients with objectively measured elevated blood pressure, FPG, or hypercholesterolemia were receiving the corresponding antihypertensive, antidiabetic, or statin treatment. These findings are in line with comprehensive reviews showing that individuals with SMI systematically receive lower‐quality medical care for cardiovascular disease and diabetes than people without psychiatric disorders, including lower rates of evidence‐based pharmacotherapy and preventive interventions [52, 53]. Taken together, and consistent with previous reports of unequal access to and quality of somatic care in this population, the comparison with population‐level benchmarks suggests that having a psychiatric disorder not only increases cardiometabolic risk but also confers an additional disadvantage in access to and implementation of guideline‐recommended somatic care [28].

Several structural and systemic factors may help explain the marked treatment gaps observed in our cohort. Prior reviews have shown that people with psychiatric disorders often navigate highly fragmented care pathways, with a lack of cooperation between different parts of healthcare systems leading to missed opportunities for diagnosis and treatment of somatic conditions [54]. In addition, stigma within healthcare settings and diagnostic overshadowing, whereby physical complaints are attributed to the psychiatric disorder, have been identified as a key mechanism underlying lower rates of investigation and evidence‐based treatment for cardiovascular and metabolic diseases in this population [55, 56]. Finally, cognitive impairment, disorganization, low health literacy, and socioeconomic disadvantage may further hinder patients' ability to navigate complex health systems and to engage in long‐term self‐management of chronic cardiometabolic conditions [57]. The partial adherence to treatment found in our sample, as measured by the MARS scale, is consistent with these overall difficulties in enrolling in care, and altogether, these structural barriers provide a plausible explanation for the substantial misalignment between cardiometabolic needs and somatic treatment that we observed in this psychiatric sample.

Three indicators of glucose metabolism were used in our study and capture partially overlapping populations. FPG ≥ 5.6 mmol/L was used to identify dysglycaemia broadly defined (in line with IDF criteria for the metabolic syndrome), HbA1c ≥ 48 mmol/mol was used to identify established diabetes, and patient‐reported diabetes reflected clinically known cases. The higher proportion of patients reporting a diabetes diagnosis (4.2%) compared with those with HbA1c‐defined diabetes (2.3% of those tested) likely reflects this distinction—well‐controlled diabetic patients can have normal biological values—combined with the selective measurement of HbA1c, which is typically performed in patients with suspected dysglycaemia or known diabetes rather than systematically across the cohort. Thyroid function appears well managed in our cohort (mean TSH 2.21 mIU/L), and 4.0% of patients reported levothyroxine treatment, consistent with the routine inclusion of thyroid testing in the assessment of patients receiving psychotropic medications.

Previous work has documented similar gaps between measured cardiometabolic abnormalities and pharmacological treatment in psychiatric populations. In the RAISE‐ETP cohort of first‐episode schizophrenia spectrum patients, Correll et al. reported particularly striking discrepancies [58], finding, for example, that 39.9% of their patients had prehypertension, 10.0% had hypertension, but only 3.6% received antihypertensive drugs. Similar gaps in somatic care have also been documented in France in the FACE‐SZ cohort [32] and in severe mental illness more broadly [52, 53]. Building on this body of work, the present study extends the documentation of these discrepancies to a large transdiagnostic sample that also includes anxiety disorders and ADHD, which together represent a substantial proportion of patients seen in psychiatric care [59]. Consistent with the established hierarchy of cardiometabolic risk in psychiatric disorders, our subgroup analyses confirmed that patients with schizophrenia carried the highest burden of measured cardiometabolic abnormalities (Table 4), while patients with ADHD and AD displayed lower prevalences across most components. Yet, despite this gradient of measured risk, the overall discordance between detected cardiometabolic abnormalities and reported pharmacological treatment remained substantial in the whole cohort, suggesting that under‐management of somatic comorbidities concerns the broader spectrum of psychiatric care, not only patients with the highest documented cardiovascular risk.

Beyond objective cardiometabolic measurements, the cohort displayed a substantial patient‐reported burden of psychotropic side effects, including weight changes and metabolic symptoms captured by the UKU subscales—particularly UKU‐I (psychic, 5.55) and UKU‐IV (other, including weight changes, 3.72). Health‐related quality of life was substantially impaired on the mental dimension (SF‐36 MCS = 30.5) and moderately impaired on the SQoL‐18 (50.1), consistent with the chronicity and severity profile of the cohort. Although our cross‐sectional design does not allow us to test causal relationships, the literature has consistently shown that adverse metabolic effects of psychotropic medications—particularly weight gain—are a major contributor to non‐adherence and treatment discontinuation in patients with severe mental illness [60]. The combination of substantial measured cardiometabolic abnormalities, patient‐reported metabolic side effects (UKU‐IV), and only moderate adherence in our cohort is consistent with this pattern and suggests that imperfect adherence—possibly driven in part by metabolic adverse effects—may contribute alongside difficulties in accessing healthcare and tolerability issues to the gap observed between measured cardiometabolic needs and reported pharmacological treatment.

Certain limitations must be acknowledged. The analyses were primarily descriptive and were not designed to identify determinants of the observed treatment‐prevalence gap, and the cross‐sectional design prevents us from drawing conclusions about the timing of the relationship between cardiometabolic disorders and treatment initiation. Data availability varied across cardiometabolic variables, with most parameters available for over 92% of the cohort except prolactin (75% of all included patients) and HbA1c (54.8%), reflecting the selective use of this measurement in clinical practice. Metformin only partially reflects the treatment of type 2 diabetes in our sample, as other available treatments, such as GLP‐1 receptor agonists, should also be considered; furthermore, it may be prescribed not only for established type 2 diabetes but also for the prevention or management of antipsychotic‐induced weight gain, which may lead to an overestimation of diabetes treatment. Given that statin prescription depends on global cardiovascular risk assessment rather than cholesterol levels alone, the observed discrepancies should solely be interpreted as a potential signal of insufficient cardiovascular risk evaluation. The single‐center nature of our study within a university psychiatric hospital also limits the generalizability of the results. Because both nonpsychiatric diagnoses and their treatments were ascertained through patient self‐report, our data are subject to potential misclassification, the extent of which may vary across patients, including those with cognitive impairment or more severe illness such as schizophrenia. Importantly, because medication data relied on patient self‐report, we cannot distinguish patients who were never offered cardiometabolic treatment from those who declined a prescription or discontinued it due to intolerance or adverse effects. The observed gap should therefore be interpreted as a discrepancy between measured needs and ongoing treatment, rather than direct evidence of under‐prescription. Linkage to prescription and dispensing registries would be needed to disentangle these mechanisms. Due to changes in the study protocol over the inclusion period, several scales were not systematically administered to all participants: quality of life was assessed using two complementary instruments—the SF‐36 and the SQoL‐18—which were not always administered to the same patients, limiting direct comparison between scales within our cohort.

## 5 Conclusion

Our study suggests a substantial gap between the medications that psychiatric patients report taking and their actual needs in terms of managing cardiometabolic risk. Elevated blood pressure and lipid abnormalities were frequent, yet only a small minority of patients reported receiving antihypertensive or lipid‐lowering medication, and metformin use was rare despite high fasting plasma glucose in a substantial proportion of the sample.

These inequalities must be given special attention; they highlight the need to consider the health of people with psychiatric disorders in all its dimensions, both mental and somatic. Our results support systematic integration of screening for cardiovascular and metabolic comorbidities in psychiatric services and highlight the need for shared care pathways between psychiatrists, general practitioners, and specialists.

Future multicentric, longitudinal protocols, large enough to compare treatment–prevalence discordance across diagnostic groups and to investigate the onset of disease and the time to somatic treatment prescription, will be needed to better understand the mechanisms underlying these findings. Such studies should also evaluate the effect of targeted interventions for patients with psychiatric disorders, such as systematic screening for prescriptions that carry a risk of metabolic syndrome or programs that include physical activity and therapeutic nutrition education, with the ultimate goal of narrowing the gap between detection and treatment and preventing avoidable cardiovascular morbidity and mortality in people receiving psychiatric care.

## Author Contributions

**Vincent Achour:** conceptualization, methodology, writing – original draft. **Melanie Faugere:** software, validation, formal analysis. **Laurent Boyer:** supervision, project administration. **Christophe Lançon:** resources, data curation, investigation. **Theo Korchia:** conceptualization, supervision, project administration, writing – review and editing. All authors contributed to the interpretation of the results, critically reviewed the manuscript, and approved the final version to be published.

## Funding

The authors have nothing to report.

## Ethics Statement

The authors assert that all procedures contributing to this work comply with the ethical standards of the relevant national and institutional committees on human experimentation and with the Helsinki Declaration of 1975, as revised in 2008.

## Consent

Written informed consent was obtained from all participants for participation and for the use of their clinical data for research purposes.

## Conflicts of Interest

The authors declare no conflicts of interest.

## Data Availability

The datasets analyzed during the current study are not publicly available due to their sensitive nature and institutional restrictions (clinical health data), but are available from the corresponding author on reasonable request, subject to approval by the institutional data access committee and the execution of a data‐sharing agreement. Only de‐identified data will be shared.
